# Volume Dependence of Hydrogen Diffusion for Sorption and Desorption Processes in Cylindrical-Shaped Polymers

**DOI:** 10.3390/polym14040756

**Published:** 2022-02-15

**Authors:** Jae Kap Jung, Kyu Tae Kim, Un Bong Baek, Seung Hoon Nahm

**Affiliations:** 1 Hydrogen Energy Materials Research Center, Korea Research Institute of Standards and Science, Daejeon 34113, Korea; ubbaek@kriss.re.kr (U.B.B.); shnahm@kriss.re.kr (S.H.N.); 2 Electricity and Magnetism Group, Korea Research Institute of Standards and Science, Daejeon 34113, Korea; ktkim@kriss.re.kr

**Keywords:** polymer, hydrogen diffusion, hydrogen uptake, sorption, desorption, numerical simulation

## Abstract

In the actual application of gas transport properties under high pressure, the important factors are sample size dependence and permeation efficiency, related to gas sorption. With a modified volumetric analysis technique, we firstly measured the overall diffusion properties and equilibrium times for reaching the saturation of hydrogen content in both hydrogen sorption and desorption processes. The measured parameters of total uptake (*C_∞_*), total desorbed content (*C*_0_), diffusion coefficient in sorption (*D_s_*), diffusion coefficient in desorption (*D_d_*), sorption equilibrium time (*t_s_*) and desorption equilibrium time (*t_d_*) of hydrogen in two polymers were determined relative to the diameter and thickness of the cylindrical-shaped polymers in the two processes. *C*_∞_ and *C*_0_ did not demonstrate an appreciable volume dependence for all polymers. The identical values of *C*_∞_ and *C*_0_ indicate the reversibility between sorption and desorption, which is interpreted by the occurrence of physisorption by sorbed hydrogen molecules. However, the measured diffusivity of the polymers was found to be increased with increasing thickness above 5 mm. Moreover, the larger *D_d_* values measured in the desorption process compared to *D_s_* may be attributed to an increased amorphous phase and volume swelling caused by increased hydrogen voids and polymer chain scission after decompression. The *t_s_* and *t_d_* were found to be linearly proportional to the square of the thickness above an aspect ratio of 3.7, which was consistent with the numerical simulations based on the solution of Fick’s law. This finding could be used to predict the *t_s_* in a polymer without any measurement, depending on the sample size.

## 1. Introduction

Sorption is an important chemical process in solid membranes, and desorption is the reverse process; both follow similar diffusion laws [1]. Gas sorption and desorption are critical factors introduced to control the permeation property in sealing applications [2,3]. The permeation efficiency is related not only to the equilibrium features but also to the kinetics of both processes under high-pressure environments [4,5]. In particular, an investigation of the saturated equilibrium and related physical stability in hydrogen permeation is essential for designing industrial equipment, reducing operating costs, and gaining insights into adsorption. The equilibrium time is also important factor for determining the appropriate exposure time to hydrogen under high pressure in cycling testing [6,7].

Permeability properties are evaluated by various methods, such as gravimetric techniques [8], the magnetic suspension balance method [9], manometric methods [10], constant pressure methods [11] and thermal desorption analysis [12]. Gas diffusion properties and sorption equilibrium time have been shown to be affected by the sample shape and volume [13,14,15,16,17]. Most evaluation techniques for gas permeation mainly monitor the pressure, volume and sample weight in measuring the cell containing the specimen to be tested. Then, the measured values are converted to the related permeation quantity through the appropriate equations. To keep the sensitivity and accuracy at a high level for the measured quantity, the sample is loaded in a limited volume of cell. This means that the evaluation techniques have limitations in determining the permeation parameters according to variations in the shape and size of the sample employed. It is also a well-known fact that the characteristic time, i.e., equilibrium time (or time lag), is dependent on the thickness or radius of the specimen used [13,14]. Furthermore, researchers have mainly focused on the effects of the physical, thermal and mechanical properties of hydrogen after pressurization and then decompression [18,19,20]. However, in situ measurements during pressurization under high pressures have rarely been conducted.

To supplement the limited research, we measured the hydrogen sorption/desorption properties in the process of pressurization/decompression, together with the size effect of the diffusion properties for cylindrical samples with different diameters and thicknesses. These were effectively investigated by employing the developed volumetric analysis technique (VAT) [21,22]. Unlike conventional techniques, the volumetric technique is a very simple and precise method to determine permeation properties, regardless of the specimen shape/dimension and gas species. Moreover, a diffusion analysis program for simulating the hydrogen transport property was upgraded for the use of various gases and different shapes (cylinder, sphere and sheet) of the specimen for both modes of emission and the remaining contents of gas. This work was conducted for rubbery polymers, such as nitrile butadiene rubber (NBR) and ethylene propylene diene monomer (EPDM). These polymers are utilized as sealing materials for O-rings in gas applications, such as hydrogen fueling stations [23]. The total uptake ($C_{\infty }$), total desorbed content ($C_{0})$, diffusion coefficient in sorption (*D_s_*), diffusion coefficient in desorption (*D_d_*), sorption equilibrium time (*t_s_*) and desorption equilibrium time (*t_d_*) of hydrogen were determined as a function of the diameter and thickness of the cylindrical-shaped polymers.

The main concern of present research was to deduce the general behavior of the sorption and desorption properties of hydrogen in two types of polymers. The equilibrium time for reaching the saturation of the hydrogen content is an important criterion applied in the related research for deciding the high-pressure exposure time for the cycling test and for designing O-ring material under high pressure. The equilibrium time was found to be directly dependent on both the volume, diffusion coefficient and aspect ratio of the cylindrical sample. The equilibrium time evaluated by experimental investigations was confirmed by performing numerical simulations based on the solution of Fick’s law. From linear correlation between the equilibrium time and specimen thickness, the sorption equilibrium time can be predicted without any further measurement.

## 2. Volumetric Analysis Technique and Measuring Principle

**Figure 1:**
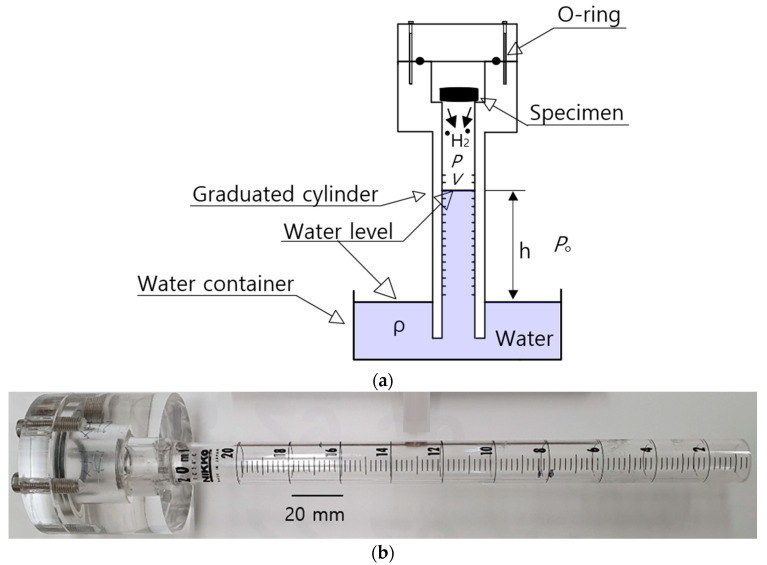
(**a**) Schematic diagram of the VAT system in which the cylinder is standing upright. The blue part indicates the distilled water filled in the water container and cylinder. (**b**) Photograph of a stretched graduated cylinder with a capacity of 20 mL.

The chemical composition of the rubber investigated in this research can be found in the literature [21,22]. The experiments were conducted after exposure and subsequent decompression. The polymer specimen was exposed to a fixed pressure of 5.75 MPa for the required residence time. After decompression to atmospheric pressure, the hydrogen gas was released from the rubber. Subsequently, the polymer from the high-pressure chamber was loaded into the graduated cylinder of the VAT system, as shown in Figure 1.

The simple VAT system shown in Figure 1a measured the released hydrogen. A graduated cylinder immersed partially in a water container collected and measured the emitted H_2_ gas with an O-ring to prevent gas leakage. Figure 1b shows a photograph of a stretched graduated cylinder with a volume capacity of 20 mL, where the volume depends on the desorbed hydrogen gas content versus pressure. The transparent acrylic graduated cylinder was employed to clearly observe the water level. The water level was precisely measured with a resolution of 0.01 mL by a digital camera or two electrodes in real time at specified time intervals for the case with a volume capacity of 10 mL.

The pressure (P) inside the graduated cylinder for hydrogen gas measurement, as shown in Figure 1a, is expressed as follows [21,22]:

(1) $$P=P_{o}-\rho gh$$

where $P_{o}$ is the outside atmosphere pressure of the cylinder, ρ is the density of distilled water in the water container, *g* is gravity and h is the height of the distilled water level, indicated by the blue in Figure 1a, inside the graduated cylinder measured from the water level in the water container. As shown in Figure 1a, the hydrogen gas released from the rubber after decompression lowers the water level of the cylinder, which is governed by the ideal gas equation, *PV* = *nRT*, where R is the gas constant (8.20544 × 10^−5^ m^3^·atm/(mol·K)), *V* is the volume inside the graduated cylinder filled with gas and *n* is the number of hydrogen gas moles. Thus, we could quantify the content of released hydrogen by measuring the change in the water level (ΔV).

The number of moles (Δn) of hydrogen gas collected inside the graduated cylinder was obtained by measuring the lowered water level (ΔV=AΔh), i.e., the volume change (ΔV) due to the hydrogen emitted from the rubber at a specified *P* and *T*, as follows [21,22]:

(2) $$\Delta n=\frac{(P_{o}-\rho gh)A\Delta h}{RT}$$

where *A* is the cross-sectional area of the cylinder and Δh is the water level lowered by the released hydrogen. The number of moles (Δn) of hydrogen gas was converted to mass concentration [C(t)] in the rubber sample as follows:

(3) $$C\left(t\right)\left[\mathrm{wt}\cdot \mathrm{ppm}\right]=\Delta n\left[\mathrm{mol}\right]\times \frac{2.016\;\left[\frac{g}{\mathrm{mol}}\right]}{m_{sample}\left[g\right]}$$

where the molar mass of hydrogen is 2.016 [g/mol] and $m_{sample}$ is the mass of the polymer. Therefore, the time-dependent mass concentration was obtained by measuring the water level change, Δh, versus the elapsed time after decompression.

Assuming that the adsorption and desorption of H_2_ is a diffusion-controlled process, the emitted H_2_ content $C_{E}\left(t\right)$ in the adsorption process and remaining H_2_ mass concentration $C_{R}\left(t\right)$ in the desorption process are expressed as (4) and (5), respectively [24,25]:

(4) $$C_{E}\left(t\right)/C_{\infty }=1-\frac{32}{\pi^{2}}\times \left[\overset{\infty }{\underset{n=0}{\sum }}\frac{exp\left\{\frac{-{\left(2n+1\right)}^{2}\pi^{2}D_{s}t}{l^{2}}\right\}}{{\left(2n+1\right)}^{2}}\right]\times \left[\overset{\infty }{\underset{n=1}{\sum }}\frac{exp\left\{-\frac{D_{s}\beta_{n}^{2}t}{\rho^{2}}\right\}}{\beta_{n}^{2}}\right]$$

(5) $$C_{R}\left(t\right)=\frac{32}{\pi^{2}}\times C_{0}\times \left[\overset{\infty }{\underset{n=0}{\sum }}\frac{exp\left\{\frac{-{\left(2n+1\right)}^{2}\pi^{2}D_{d}t}{l^{2}}\right\}}{{\left(2n+1\right)}^{2}}\right]\times \left[\overset{\infty }{\underset{n=1}{\sum }}\frac{exp\left\{-\frac{D_{d}\beta_{n}^{2}t}{\rho^{2}}\right\}}{\beta_{n}^{2}}\right]$$

Equations (4) and (5) are the solutions to Fick’s second diffusion law for a cylindrical-shaped specimen. A constantly uniform hydrogen concentration is initially maintained and the cylindrical surfaces are kept at a constant concentration. In Equations (4) and (5), l is the thickness of the cylindrical rubber sample, ρ  is the radius and $\beta_{n}$ is the root of the zero-order Bessel function.

In Equation (4), $C_{\infty }$ is the saturated hydrogen mass at an infinitely long time, i.e., the total emitted mass concentration or hydrogen uptake in the sorption process, and in Equation (5), $C_{0}$ is the remaining mass concentration at t = 0 in the desorption process—that is, the total desorption content. In addition, $D_{s}$ and $D_{d}$ are the diffusion coefficients of sorption and desorption, respectively.

To analyze the time-varying mass concentration data in the form of a multiexponential function, we used a diffusion analysis program developed using Visual Studio to calculate $D_{s}$, $D_{d},$ $C_{\infty }$ and $C_{0}$ in Equations (4) and (5) based on the least-squares regression method [22,26].

## 3. Sequence for Measuring Diffusion in Sorption and Desorption

The elapsed time after decompression was counted from the moment (*t* = 0) at which the high-pressure chamber was reduced to atmospheric pressure. The released content obtained from the measurement was regarded as the sorption content of hydrogen because all entering hydrogen was entirely emitted.

**Figure 2:**
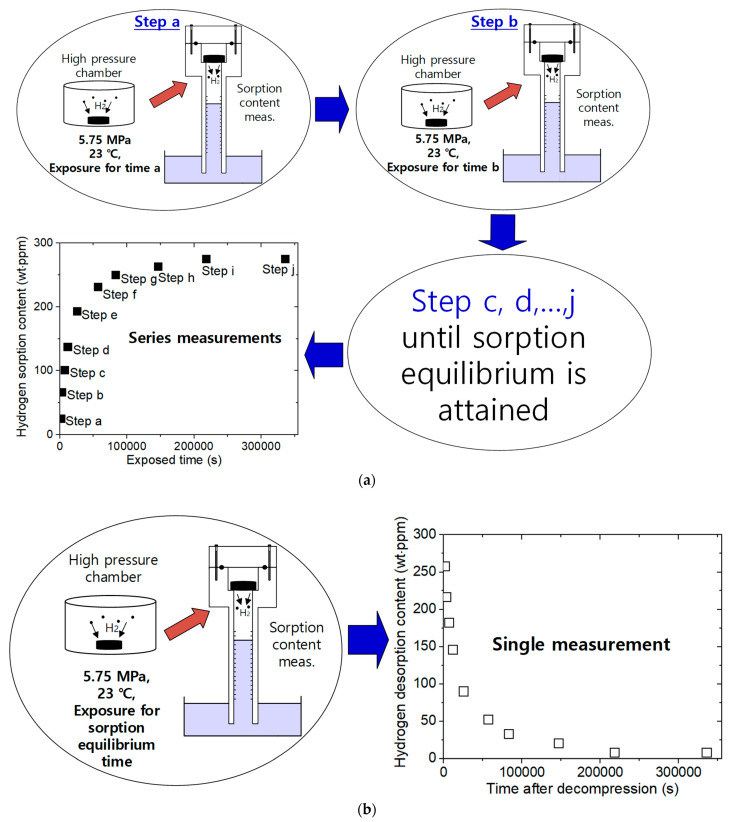
(**a**) Clockwise sequence for measuring the diffusion parameter in the sorption process. The measurement was performed by VAT after loading the rubber exposed to a high-pressure chamber inside the graduated cylinder. The sorption contents were measured as a function of the residence time (exposed time, a, b,…, j) of the specimen in the high-pressure chamber. This array of measurements corresponded to a series measurement. (**b**) Sequence for measuring the diffusion parameter in the desorption process. The measurement was performed by VAT after loading the rubber exposed to a high-pressure chamber inside the graduated cylinder. The desorption contents were determined by a single measurement after the exposure of the specimen for equilibrium time.

The sequence for measuring the diffusion parameters of sorption and desorption is displayed in Figure 2a,b, respectively. The hydrogen sorption contents were measured as a function of the residence time (exposed time) of specimens exposed to a high-pressure chamber using VAT. The sorption quantity in units of hydrogen mass concentration in Equation (3) versus the elapsed time obtained by VAT after decompression exposed at a single residence time, a, is shown in step a of Figure 2a. As a result of this measurement, $C_{0}\left(t=a\right)$ at time a was obtained by Equation (5). As shown in step b of Figure 2a, $C_{0}\left(t=b\right)$ at time b was obtained by Equation (5) after decompression for exposure at residence time b. The $C_{0}$ varying residence times (time a, b, …, j) were collected until the hydrogen sorption equilibrium was reached. Thus, the sorption data array was obtained from a series of measurements of desorption after subsequent exposure times. From the $C_{0}$ versus the exposed time shown in Figure 2a, the *C*_∞_ and *D_s_* of hydrogen were determined by applying the diffusion analysis program based on Equation (4) to the measured results. The sequence for measuring the sorption properties is quite time-consuming.

In the case of the desorption process in Figure 2b, the hydrogen desorption content and diffusivity were determined from a single measurement after decompression for exposure to a sufficiently long equilibrium sorption time for the specimens under a high-pressure chamber. From the desorption data shown in Figure 2b, we determined *C*_0_ and *D_d_* by fitting with Equation (5). Thus, desorption is an easier process to complete because the measurement requires one step with one sample.

## 4. Results and Discussion

**Figure 3:**
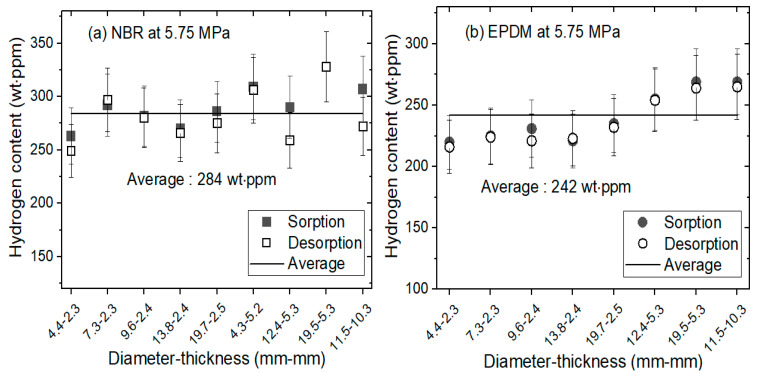
Total hydrogen sorption ($C_{\infty }$) and desorption content ($C_{0})$ versus sample volume for cylindrically-shaped (**a**) NBR and (**b**) EPDM exposed to 5.75 MPa and 296 K. $C_{\infty }$ and $C_{0}$ were obtained by the sequences in Figure 2a,b, respectively, with the application of the diffusion analysis program.

The hydrogen diffusion properties in the two processes were measured according to the sorption and desorption procedures shown in Figure 2. Figure 3 shows the investigation results of hydrogen sorption ($C_{\infty })$ and desorption content $(C_{0}$) for cylindrically-shaped NBR and EPDM with different diameters and thicknesses. The values of $C_{\infty }$ and $C_{0}$ were analyzed by Equations (4) and (5), respectively, using the diffusion analysis program.

The general behavior illustrated in Figure 3a,b for the NBR and EPDM, respectively, is as follows: both the total sorption content, $C_{\infty },$ and the total desorption content, $C_{0}$, in each rubber were consistent regardless of the sample diameter and thickness. As shown in Figure 3a, the average $C_{\infty }\;\mathrm{and}\;C_{0}$ values (284 wt·ppm) in the NBR were equivalent within the uncertainty range. As shown in Figure 3b, the average $C_{\infty }\;\mathrm{and}\;C_{0}$ values (242 wt·ppm) in the EPDM were also equivalent within the uncertainty range. This finding indicates that the sorption and desorption processes of hydrogen in the NBR and EPDM are reversible because physisorption, rather than chemisorption, occurs by introducing hydrogen. This result is consistent with previous reports showing that high-pressure hydrogen exposure does not cause any chemical structure changes in NBR by nuclear magnetic resonance analysis [23,27]. The reversible adsorption phenomenon of hydrogen has been commonly observed in the literature [24,25]. In particular, in hydrogen storage materials, such as porous carbon, reversibility is defined as its ability to retain its storage capacity during repeated hydrogen charging and discharging in long-term cycling stability, and it represents an important quality.

Physisorption is a reversible process with weak adsorption energy (20–40 kJ/mol) governed by van der Waals forces, while chemisorption is an irreversible chemical process with strong adsorption energy above 40–200 kJ/mol bounded by covalent or ionic bonding. In our diffusivity measurement, the activation energy E_a_ for the EPDM specimen obtained through temperature dependence was found to be in the range of 20–35 kJ/mol. Thus, we assume hydrogen adsorption–desorption is governed by a physisorption process in nature. In addition, in previous similar research on polymers [23,27] it was reported that the hydrogen desorbed did not affect the chemical structure.

**Figure 4:**
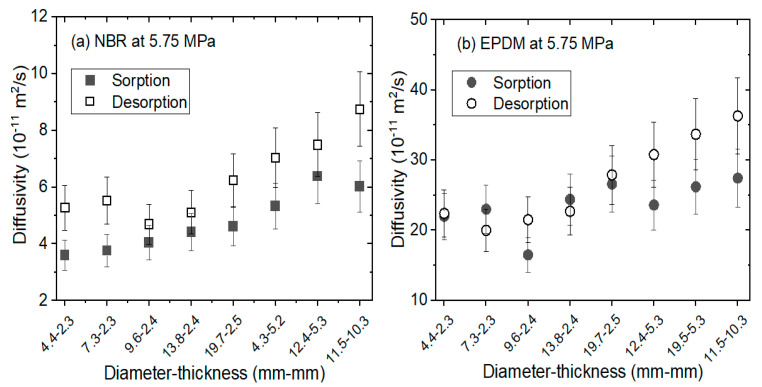
Comparison of the diffusion coefficients ($D_{s}$ and $D_{d})$ versus sample volume for cylindrically-shaped (**a**) NBR and (**b**) EPDM exposed to 5.75 MP and 296 K. $D_{s}$ and $D_{d}$ were obtained by the sequences in Figure 2a,b, respectively, with the application of the diffusion analysis program.

Meanwhile, the hydrogen diffusivity versus the diameter and thickness of the sample for the two processes are shown in Figure 4. The values of *D_s_* and *D_d_* were analyzed by Equations (4) and (5), respectively, with the diffusion analysis program. The diffusivity in the sorption and desorption processes displayed volume dependence above a thickness of 5 mm. The thickness of the sample during diffusion was a more sensitive factor than the diameter. The investigation results in Figure 4 indicate that the diffusion coefficient in desorption $D_{d}$ was higher than $D_{s}$ in the sorption process for both rubbers. The difference in diffusion observed between the two processes indicates that the sorption and desorption processes are different from each other. The faster desorption may be significantly attributed to the increase in hydrogen diffusion due to rapid decompression yielding expanded hydrogen voids and volume swelling. Furthermore, hydrogen penetration causes scission of the polymer chain and diffusion takes place in the amorphous region, as indicated in the literature [26,27].

**Figure 5:**
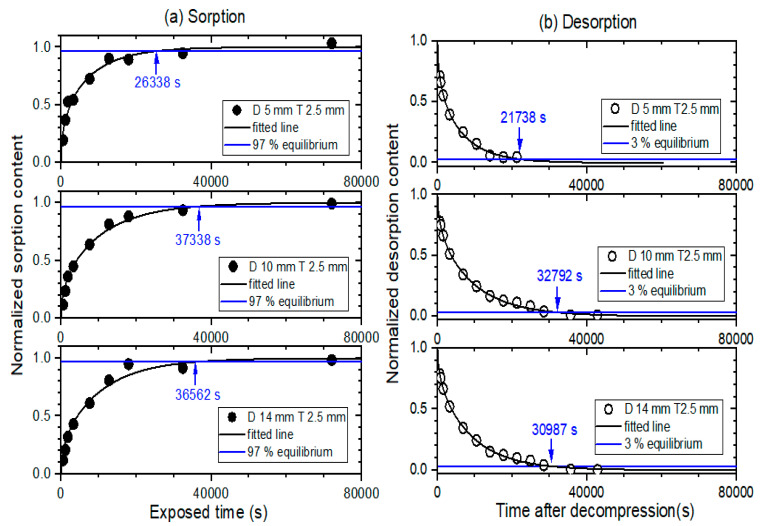
(**a**) Normalized sorption content versus exposed time and (**b**) desorption content versus time after decompression for cylindrically-shaped NBR with a diameter of 5 mm and thickness of 2.5 mm, a diameter of 10 mm and thickness of 2.5 mm, and a diameter of 14 mm and thickness of 2.5 mm. D and T represent the diameter and thickness, respectively. The normalized sorption and desorption contents were obtained by the sequences in Figure 2a,b, respectively. The values indicated by the arrows are the equilibrium time measured in the sorption and desorption process.

Furthermore, the sorption and desorption curves had a multi-exponential form with varying time. Thus, the equilibrium time in the two processes was defined as the time at which the hydrogen content reached 97% of the total sorption content, i.e., *C*(*t*) = 0.97 for $\;C_{\infty }$, and 3% of the total desorption content, i.e., *C*(*t*) = 0.03 for $C_{0}$. Figure 5a,b display the curves of normalized sorption content versus exposed time and normalized desorption content versus time after decompression, respectively, with varying diameters at a fixed thickness of 2.5 mm for NBR. As shown in Figure 5a, the corresponding sorption equilibrium times (blue arrow) obtained for NBR were 26,338 s for a diameter of 5 mm and thickness of 2.5 mm; 37,338 s for a diameter of 10 mm and thickness of 2.5 mm; and 36,562 s for a diameter of 14 mm and thickness of 2.5 mm. Meanwhile, the desorption equilibrium times (blue arrow) determined for NBR, as shown in Figure 5b, were 21,738 s for a diameter of 5 mm and thickness of 2.5 mm; 32,792 s for a diameter of 10 mm and thickness of 2.5 mm; and 30,987 s for a diameter of 14 mm and thickness of 2.5 mm.

**Figure 6:**
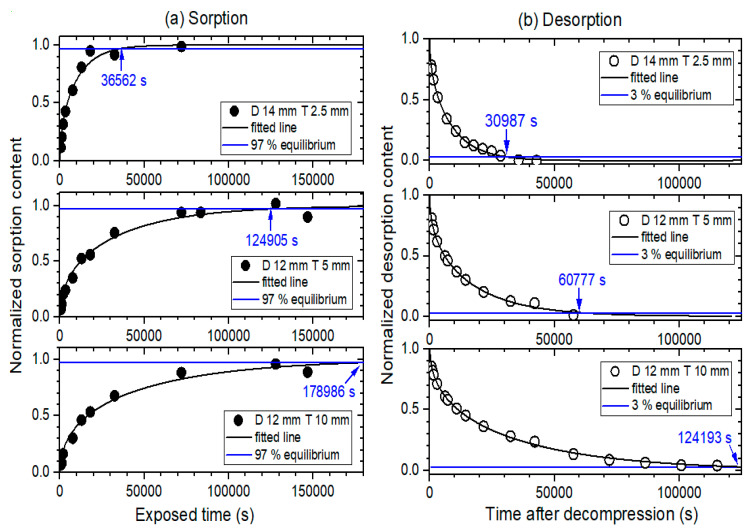
(**a**) Normalized sorption content versus exposed time and (**b**) normalized desorption content versus time after decompression for cylindrically-shaped NBR with a diameter of 14 mm and thickness of 2.5 mm, a diameter of 12 mm and thickness of 5 mm, and a diameter of 12 mm and thickness of 10 mm. D and T represent the diameter and thickness, respectively. The normalized sorption and desorption contents were obtained by the sequences in Figure 2a,b, respectively. The values indicated by the arrows are the equilibrium time measured in the sorption and desorption process.

Figure 6a,b show the normalized sorption and desorption curves versus time, respectively, with varying thicknesses and similar diameters for NBR. The corresponding sorption equilibrium times (blue arrow) obtained for NBR were 36,562 s for a diameter of 14 mm and thickness of 2.5 mm; 124,905 s for a diameter of 12 mm and thickness of 5 mm; and 178,986 s for a diameter of 12 mm and thickness of 10 mm. The desorption equilibrium times (blue arrow) obtained for NBR were 30,987 s for a diameter of 14 mm and thickness of 2.5 mm; 60,777 s for a diameter of 12 mm and thickness of 5 mm; and 124,193 s for a diameter of 12 mm and thickness of 10 mm. EPDM also displayed a similar volume dependence for equilibrium time as NBR. The equilibrium time in EPDM was faster compared to that measured in NBR because of its faster diffusivity, as shown in Figure 4.

**Figure 7:**
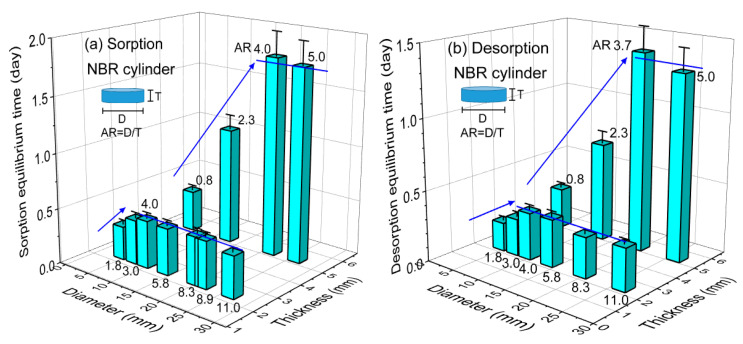
Three-dimensional plot of (**a**) sorption and (**b**) desorption equilibrium time versus volume for NBR. The number at the top of the bar indicates the aspect ratio (AR = D/T) of the cylindrical rubber. D and T indicate the diameter and thickness, respectively, of the cylindrical-shaped specimen. The blue slanted lines with arrows indicate the linear increase in equilibrium time versus thickness. The blue horizontal lines indicate constant equilibrium time versus diameter.

The equilibrium times of sorption and desorption measured for cylindrically-shaped NBR are visualized in Figure 7a,b, respectively, through a three-dimensional plot of the corresponding sorption and desorption equilibrium time versus the diameter and thickness for NBR. The aspect ratio (AR = D/T) is defined as the diameter (D) with regard to the thickness (T) of the cylindrical sample. In the case of a thickness of 2.3 mm, as shown in Figure 7a,b, the sorption and desorption equilibrium time increased with increasing diameter up to an AR of 3.7 (slanted blue arrow) and was nearly constant above an AR of 3.7 (horizontal blue line). In the case of a thickness of 5.3 mm, as shown in Figure 7a,b, a similar diameter dependence to that observed with a thickness of 2.3 mm was also found. In other words, the sorption and desorption equilibrium time increased with increasing diameter up to an AR of 3.7 and was nearly constant above an AR of 3.7. Meanwhile, the sorption and desorption equilibrium time increased with increasing thickness at a constant diameter. As shown in Figure 4, the change in equilibrium time versus thickness is steeper than that observed for diameter.

**Figure 8:**
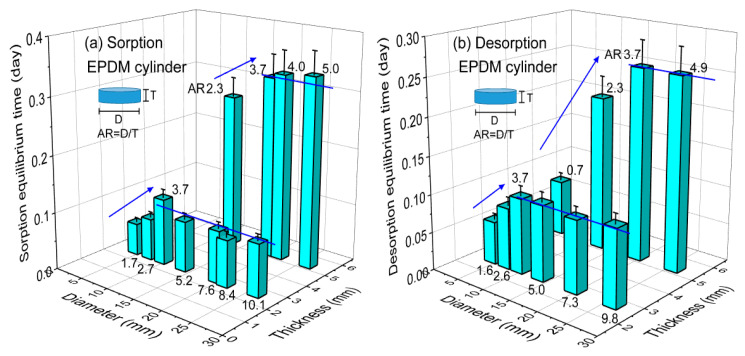
Three-dimensional plot of (**a**) sorption and (**b**) desorption equilibrium time versus volume for EPDM. The number at the top of the bar indicates the aspect ratio (AR = D/T) of the cylindrical rubber. D and T indicate the diameter and thickness, respectively, of the cylindrical-shaped specimen. The blue slanted lines with arrows indicate the linear increase in equilibrium time versus thickness. The blue horizontal lines indicate constant equilibrium time versus diameter.

Similar to the NBR sample, we also visualized the equilibrium time of sorption and desorption for cylindrically-shaped EPDM with varying diameters or thicknesses. Figure 8a,b show the three-dimensional plot of the corresponding sorption and desorption equilibrium time versus volume for EPDM. In the case of a thickness of 2.3 mm, as shown in Figure 8a,b, the sorption and desorption equilibrium time increased with increasing diameter up to an AR of 3.7 (slanted blue arrow) and was nearly constant above an AR of 4.0 (horizontal blue line). In the case of a thickness of 5.3 mm, as shown in Figure 8a,b, a similar diameter dependence was also found. Meanwhile, the sorption and desorption equilibrium time increased with increasing thickness at a fixed diameter. The change in equilibrium time versus thickness steepened compared with that observed for diameter.

**Figure 9:**
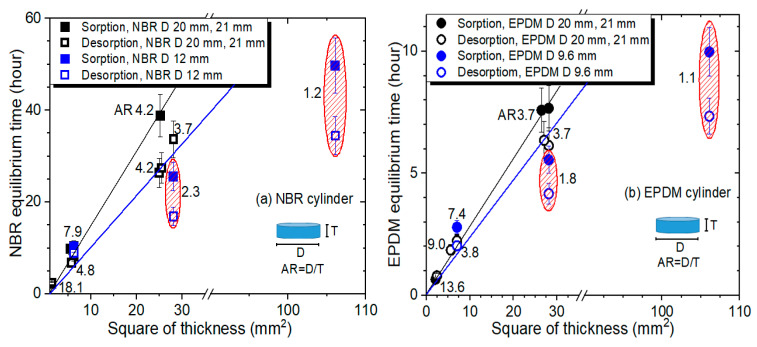
Saturation time for the sorption equilibrium and desorption equilibrium versus the square of the thickness for (**a**) NBR and (**b**) EPDM. The number indicates the aspect ratio (AR) of the cylindrical rubber. D indicates diameter of the cylindrical specimen.

After the investigations shown in Figure 7 and Figure 8, we focused on the effect of the sensitive thickness dependence on the equilibrium time of both the sorption and desorption processes for the two rubbers. Figure 9a,b depict the saturation time for the sorption/desorption equilibrium time versus the square of the thickness for NBR and EPDM cylindrical-shaped rubber, respectively. The experimental observations indicated that the thicker the sample was, the longer the time to reach hydrogen equilibrium saturation. The investigations clearly demonstrated a linear correlation between the saturation time and the square of the thickness above an AR of 3.7 for both specimens. In Figure 9a, the black line indicates the linear relation between the sorption equilibrium time and the square of the thickness for NBR. In Figure 9a, the blue line indicates the linear relation between the desorption equilibrium time and the square of the thickness for NBR. In Figure 9b, the black (blue) line indicates the linear relation between the sorption (desorption) equilibrium time and the square of the thickness for EPDM. The reciprocal slope indicates the diffusion coefficient. The faster diffusion coefficient for EPDM than NBR is attributed to the short equilibrium time, which corresponds to a small slope in the equilibrium time with regard to the square of the thickness.

According to Equations (4) and (5), the characteristic time is proportional to the squared thickness in the case where diffusivity is constant. This is a well-known fact for those cases with constant diffusivity. However, because the size-dependent diffusivity was observed, it must be confirmed experimentally whether the linearity between the equilibrium time and squared thickness is still true or not. If the experimental results comply with the linearity, the equilibrium time for various different thickness can be predicted from the linear relationship without further measurement. The experimental finding, i.e., linearity, is still applied, even though the variation of diffusivity still exists.

In addition, the linearity deviated below an AR of 3.7 for both NBR and EPDM, as shown in ellipses with red oblique lines in Figure 9.

**Figure 10:**
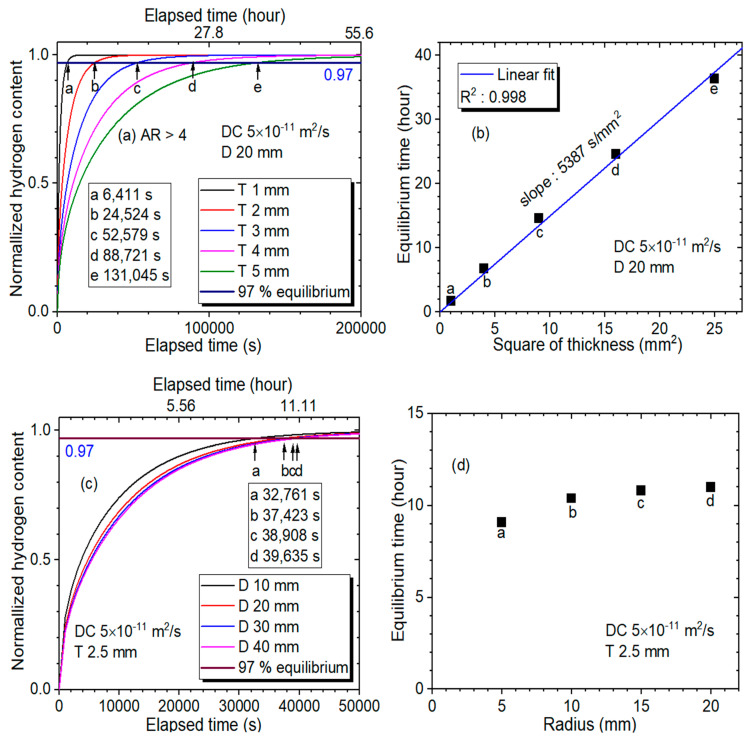
Numerical simulation for (**a**) normalized hydrogen content versus elapsed time with different thicknesses, (**b**) linear correlation between equilibrium time and square of thickness, (**c**) normalized hydrogen content versus elapsed time with different diameters, and (**d**) equilibrium time versus radius with a DC of 5 × 10^−11^ m^2^/s and thickness of 2.5 mm. D and T indicate the diameter and thickness of the cylindrical-shaped specimen, respectively. a, b, c, d and e are equilibrium time. DC—diffusion coefficient.

To verify the linearity observed between the equilibrium time and the square of the thickness above an aspect ratio of 3.7, a numerical simulation based on the solution of Fick’s law was conducted for the rubbers. Figure 10a presents the normalized hydrogen content for different thicknesses (T) with a diffusion coefficient (DC) of 5 × 10^−11^ m^2^/s and a diameter (D) of 20 mm. Panels a, b, c, d and e indicate the equilibrium times for thicknesses of 1 mm, 2 mm, 3 mm, 4 mm and 5 mm, respectively. Figure 10b was plotted from the simulation result of Figure 10a. The distinct linear dependence between the equilibrium time and the square of the thickness with a correlation coefficient R^2^ = 0.998 was demonstrated, which was consistent with the experimental investigation, as shown in Figure 9a,b.

Moreover, Figure 10c shows the numerical simulation of normalized hydrogen content for different diameters with a DC of 5 × 10^−11^ m^2^/s and thickness (T) of 2.5 mm for the case with an AR of 3.7. Panels a, b, c and d indicate the equilibrium time for diameters of 10 mm, 20 mm, 30 mm and 40 mm, respectively. The simulation result replotted in Figure 10d indicates that the equilibrium time slightly increased with increasing radius.

## 5. Conclusions

By utilizing both the volumetric analysis technique and the established procedure for measuring the sorption parameters during pressurization, we characterized the sorption and desorption properties of hydrogen in two cylindrical rubbery polymers. The hydrogen content, diffusion coefficient and equilibrium time versus the sample volume were obtained for both the sorption and desorption processes. Volume dependence was not observed for *C*_0_ and $C_{\infty }$, whereas it was observed for *D_s_* and *D_d_*. The volume effect demonstrates that the thickness during diffusion was a more critical factor than the diameter of the specimen. The reversibility in hydrogen content observed between the sorption and desorption processes was ascribed to the occurrence of physisorption, rather than chemisorption, by the introduction of hydrogen.

The sorption and desorption equilibrium time was mainly affected by the following important factors: diffusion coefficient, sample thickness and aspect ratio of the sample employed. An aspect ratio of approximately 3.7 was a critical region where the equilibrium time was proportional to the square of the thickness above it but not below it. The aspect ratio of 3.7 was also a critical region whether the equilibrium time was nearly unaffected by a diameter above it but not below it.

The equilibrium time for polymers with different thicknesses at known diffusivities could be estimated from the linear relationship without experimental measurements, which was confirmed by predictive numerical simulations. Therefore, numerical simulations can be performed to predict the sorption equilibrium time for polymers and metals in the form of a cylinder, sheet or sphere.

## Data Availability

The data used to support the findings of this study are available from the corresponding author upon request.
